# The relationship between genetic liability, childhood maltreatment, and IQ: findings from the EU-GEI multicentric case–control study

**DOI:** 10.1007/s00127-023-02513-0

**Published:** 2023-06-19

**Authors:** Lucia Sideli, Monica Aas, Diego Quattrone, Daniele La Barbera, Caterina La Cascia, Laura Ferraro, Luis Alameda, Eva Velthorst, Giulia Trotta, Giada Tripoli, Adriano Schimmenti, Andrea Fontana, Charlotte Gayer-Anderson, Simona Stilo, Fabio Seminerio, Crocettarachele Sartorio, Giovanna Marrazzo, Antonio Lasalvia, Sarah Tosato, Ilaria Tarricone, Domenico Berardi, Giuseppe D’Andrea, EU-GEI WP2 Group, Silvia Amoretti, Álvaro Andreu-Bernabeu, Grégoire Baudin, Stephanie Beards, Chiara Bonetto, Elena Bonora, Bibiana Cabrera, Angel Carracedo, Thomas Charpeaud, Javier Costas, Doriana Cristofalo, Pedro Cuadrado, Manuel Durán-Cutilla, Aziz Ferchiou, David Fraguas, Nathalie Franke, Flora Frijda, Paz Garcia-Portilla, Javier González Peñas, Kathryn Hubbard, Stéphane Jamain, Estela Jiménez-López, Marion Leboyer, Cloe Llorente, Gonzalo López Montoya, Esther Lorente-Rovira, Covadonga M. Díaz-Caneja, Camila Marcelino Loureiro, Mario Matteis, Elles Messchaart, Ma Dolores Moltó, Gisela Mezquida, Carmen Moreno, Roberto Muratori, Juan Nacher, Mara Parellada, Marta Rapado-Castro, Mirella Ruggeri, Jean-Romain Richard, José Juan Rodríguez Solano, Pilar A. Sáiz, Teresa Sánchez-Gutierrez, Emilio Sánchez, Franck Schürhoff, Marco Seri, Rosana Shuhama, Fabian Termorshuizen, Anne-Marie Tronche, Daniella van Dam, Elsje van der Ven, Celso Arango, Manuel Arrojo, Miguel Bernardo, Julio Bobes, Julio Sanjuán, Jose Luis Santos, Paulo Rossi Menezes, Cristina Marta Del-Ben, Hannah E. Jongsma, Peter B. Jones, James B. Kirkbride, Pierre-Michel Llorca, Andrea Tortelli, Baptiste Pignon, Lieuwe de Haan, Jean-Paul Selten, Jim Van Os, Bart P. Rutten, Richard Bentall, Marta Di Forti, Robin M. Murray, Craig Morgan, Helen L. Fisher

**Affiliations:** 1 Department of Human Science, LUMSA University, Piazza delle Vaschette, 101, 00193 Rome, Italy; 2 Department of Psychosis Studies, Institute of Psychiatry, Psychology & Neuroscience, King’s College London, London, England; 3 Department of Biomedicine, Neuroscience, and Advanced Diagnostic, University of Palermo, Palermo, Italy; 4 Social, Genetic and Developmental Psychiatry Centre, Institute of Psychiatry, Psychology and Neuroscience, King’s College London, London, England; 5 NORMENT Centre for Psychosis Research, Oslo University Hospital and University of Oslo, Oslo, Norway; 6 Department of Behavioural Sciences, OsloMet, Oslo Metropolitan University, Oslo, Norway; 7 Centro Investigacion Biomedica en Red de Salud Mental (CIBERSAM), Seville, Spain; 8 Instituto de Biomedicina de Sevilla (IBIS), Hospital Universitario Virgen del Rocio, Departamento de Psiquiatria, Universidad de Sevilla, Seville, Spain; 9 Service of General Psychiatry, Treatment and Early Intervention in Psychosis Program, Lausanne University Hospital (CHUV), Lausanne, Switzerland; 10 Department of Research, Mental Health Service Organization ‘GGZ Noord-Holland-Noord’, Hoorn, The Netherlands; 11 Department of Health Promotion, Mother and Child Care, Internal Medicine and Medical Specialties, University of Palermo, Palermo, Italy; 12 Faculty of Human and Social Sciences, UKE - Kore University of Enna, Enna, Italy; 13 Department of Health Services and Population Research, Institute of Psychiatry, Psychology & Neuroscience, King’s College London, London, England; 14 ESRC Centre for Society and Mental Health, King’s College London, London, UK; 15 Department of Mental Health and Addiction Services, ASP Crotone, Crotone, Italy; 16 Section of Psychiatry, Department of Neuroscience, Biomedicine and Movement, University of Verona, Verona, Italy; 17 Department of Medical and Surgical Sciences, Alma Mater Studiorum - Bologna University, Bologna, Italy; 18 Department of Biomedical and NeuroMotor Sciences, Psychiatry Unit, Alma Mater Studiorum - Bologna University, Bologna, Italy; 19 Institute of Psychiatry and Mental Health, Department of Child and Adolescent Psychiatry, Hospital General Universitario Gregorio Marañón, School of Medicine, Universidad Complutense, ISGM, CIBERSAM, Madrid, Spain; 20 Department of Psychiatry, Psychiatric Genetic Group, Instituto de Investigación Sanitaria de Santiago de Compostela, Complejo Hospitalario Universitario de Santiago de Compostela, Santiago, Spain; 21 Barcelona Clinic Schizophrenia Unit, Hospital Clinic, Departament de Medicina, Institut de Neurociències (UBNeuro), Universitat de Barcelona (UB), Institut d’Investigacions Biomèdiques, August Pi I Sunyer (IDIBAPS), Barcelona, Spain; 22 Centro de Investigación Biomédica en Red de Salud Mental (CIBERSAM), Instituto de Salud Carlos III, Madrid, Spain; 23 Department of Medicine, Psychiatry Area, School of Medicine, Universidad de Oviedo, ISPA, INEUROPA, Centro de Investigación Biomédica en Red de Salud Mental (CIBERSAM), Oviedo, Spain; 24 Department of Psychiatry, School of Medicine, Universidad de Valencia, Centro de Investigación Biomédica en Red de Salud Mental, Valencia, Spain; 25 Department of Psychiatry, Hospital “Virgen de La Luz”, Cuenca, Spain; 26 Faculdade de Medicina, Universidade de São Paulo, São Paulo, Brazil; 27 Faculdade de Medicina de Ribeirão Preto, Universidade de São Paulo, São Paulo, Brazil; 28 PsyLife Group, Division of Psychiatry, University College London, London, England; 29 Department of Psychiatry, University of Cambridge, Cambridge, England; 30 CAMEO Early Intervention Service, Cambridgeshire and Peterborough National Health Service Foundation Trust, Cambridge, England; 31 EA 7280 Npsydo, Université Clermont Auvergne, Clermont-Ferrand, France; 32 Establissement Public de Santé, Maison Blanche, Paris, France; 33 AP-HP, Groupe Hospitalier “Mondor”, Pôle de Psychiatrie, Créteil, France; 34 Institut National de la Santé et de la Recherche Médicale, U955, Créteil, France; 35 Fondation FondaMental, Créteil, France; 36 Early Psychosis Section, Department of Psychiatry, Amsterdam UMC, Amsterdam, The Netherlands; 37 Institute for Mental Health, GGZ Rivierduinen, Leiden, The Netherlands; 38 Department of Psychiatry and Neuropsychology, School for Mental Health and Neuroscience, Maastricht University Medical Centre, Maastricht, The Netherlands; 39 Department Psychiatry, Utrecht University Medical Centre, Utrecht, The Netherlands; 40 Department of Psychology, University of Sheffield, Cathedral Court, 1 Vicar Lane, Sheffield, S1 2LT UK

**Keywords:** Childhood adversity, Cognition, Family history of psychosis, First episode, Polygenic risk score, Psychosis

## Abstract

This study investigated if the association between childhood maltreatment and cognition among psychosis patients and community controls was partially accounted for by genetic liability for psychosis. Patients with first-episode psychosis (*N* = 755) and unaffected controls (*N* = 1219) from the EU-GEI study were assessed for childhood maltreatment, intelligence quotient (IQ), family history of psychosis (FH), and polygenic risk score for schizophrenia (SZ-PRS). Controlling for FH and SZ-PRS did not attenuate the association between childhood maltreatment and IQ in cases or controls. Findings suggest that these expressions of genetic liability cannot account for the lower levels of cognition found among adults maltreated in childhood.

**Supplementary Information:**

The online version contains supplementary material available at 10.1007/s00127-023-02513-0.

## Introduction

Literature suggests that the association between childhood maltreatment (i.e., abuse and neglect) and later cognitive functioning might be weaker among people with psychosis compared with unaffected controls [1]. Indeed, in a previous study on the EU-GEI sample of patients with first-episode psychosis (FEP) and community controls, we found that the association between reported exposure to maltreatment in childhood and a lower Intelligence Quotient (IQ) in adulthood was weaker among patients than controls [2]. This difference might be due to the confounding effect of other risk factors associated with both childhood maltreatment and cognition that are more prevalent among people with psychosis than unaffected controls, such as genetic liability to psychotic disorders [3–7]. Moreover, among individuals with psychosis, the impact of environmental risk factors (such as maltreatment) on cognition might be reduced by a floor effect related to genetic liability and neurodevelopmental alterations [8, 9].

Only a few studies have explored the relationship between genetic liability, childhood maltreatment, and cognition among people experiencing psychosis. For instance, the NAPLS-3 study found that among youth at clinically high risk for psychosis, family history of psychosis (FH) was associated with both greater trauma exposure and lower IQ compared with no-FH [10]. Polygenic risk scores for schizophrenia (SZ-PRS) [11, 12] have been associated with lower IQ in the general population [9, 13–15], but the findings have not consistently been replicated among people with schizophrenia [16–18]. Although SZ-PRS has also shown a modest association with childhood maltreatment [19], to our knowledge no study has investigated the role of genetic liability for psychosis in the association between childhood maltreatment and cognition.

Therefore, in this study, we aimed to investigate whether the association between childhood maltreatment (overall maltreatment, and abuse and neglect separately) and IQ could be partially accounted for by genetic liability (defined as either having FH or based on SZ-PRS). We hypothesised that genetic liability to psychosis would contribute to attenuating the association between reported exposure to maltreatment in childhood and lower IQ in adulthood among FEP patients but not among controls.

## Methods

### Participants and procedure

The EU-GEI study is a multi-centre case–control study carried out between May 2010 and April 2015 in five European countries and Brazil [20, 21]. The study was approved by the Internal Review Boards of the study centres and conducted according to International ethical standards.

People with FEP were individuals aged 18–64, living in the study catchment areas, who approached mental health services for the first time for a primary diagnosis of a psychotic disorder (ICD-10 diagnoses: F20–F33) during the study period. Community controls were individuals who were representative of the same population as the cases, and who had never been referred or treated for psychotic disorders. Information about inclusion and exclusion criteria, sampling methods, and diagnostic procedures, have been detailed elsewhere [21, 22] and are described in the Supplementary Materials.

### Measures

Cognitive functioning was estimated from IQ, assessed using an abbreviated, psychometrically robust version of the Wechsler Adult Intelligence Scales (WAIS-III) [23–25].

Genetic liability was defined as: (a) family history of psychosis (FH) and (b) polygenic risk scores for schizophrenia (SZ-PRS). FH was defined as having a first-degree relative affected by a psychotic disorder and assessed using the Family Interview for Genetic Studies (FIGS) [26]. SZ-PRSs were calculated only on the sub-sample of European ancestry participants, using the genotype procedure, population stratification, and SZ-PRS calculation as described previously [27–29] and in the Supplementary Materials.

Self-reported exposure to childhood maltreatment was assessed using the Childhood Trauma Questionnaire (CTQ) [30]. Mean ratings for physical, sexual, and emotional abuse items were used to create a childhood abuse score, mean ratings for physical and emotional neglect items were used to create a childhood neglect score, and the mean of all items was used to create an overall childhood maltreatment score. Consistent with previous studies [2, 8], these variables were dichotomised using the 80th percentile of the control group’s score as the cut-off value. These cut-offs were identical to those used in the original study [2]. In the subset with SZ-PRS data, the empirically derived cut-off used to calculate dichotomic measures of childhood abuse (present/absent) was slightly lower than that used in the original study (1.33 vs. 1.40, respectively).

### Analyses

General linear regression models stratified for cases and controls were conducted to explore whether the association between overall childhood maltreatment (and separately childhood abuse and childhood neglect) and IQ (model 1), was attenuated by the inclusion in the model of FH or SZ-PRS (model 2). The analyses were then additionally controlled for sex, age, ethnicity, education, study country, and lifetime cannabis use (model 3); and then also for current use of antipsychotics (model 4) only in the FEP group. Measurement of covariates is described in the Supplementary Materials. Results are reported as regression coefficients (*B*) and standardized regression coefficients (*β*). Analyses were run using the Statistical Package for the Social Sciences (SPSS) program version 27.0.

## Results

### Association between childhood maltreatment, FH, and IQ

Information about FH was available for 91.1% of the FEP cases (*n* = 755) and 95% of controls (*n* = 1219) from the original sample (see Supplementary Table 1 for sample demographics). Associations between case status and (i) childhood maltreatment and (ii) lower IQ are reported in Supplementary Table 2 and were similar to those detected in the full sample [2].

In the crude models, IQs were on average substantially lower among community controls exposed to childhood maltreatment (*B* =  − 5.29 [− 7.86, − 2.72], *p* < 0.001), childhood abuse (*B* =  − 6.27 [− 8.89, − 3.65], *p* < 0.001), and childhood neglect (*B* =  − 5.16 [− 7.69, − 2.63], *p* < 0.001), compared with unexposed controls. Among people with FEP, only childhood neglect was associated with lower IQ (*B* =  − 2.90 [− 5.50, − 0.29], *p* = 0.029) (see Table 1 & Supplementary Table 3)—and to a more modest extent than among controls.

**Table 1:** Association between childhood maltreatment and IQ controlled for family history of psychosis and social and clinical confounders

Unstandardized models	Model 1			Model 2			Model 3			Model 4		
	*B*	95% CI	*p*	*B*^a^	95% CI	*p*	*B*^a+b^	95% CI	*p*	*B*^a+b+c^	95% CI	*p*
Controls	*N* = 1219			*N* = 1219			*N* = 1216					
Overall maltreatment*	** − 5.29**	** − 7.86, − 2.72**	** < 0.001**	** − 5.25**	** − 7.82; − 2.68**	** < 0.001**	** − 2.30**	** − 4.55; − 0.54**	**0.045**			
Abuse	** − 6.27**	** − 8.89, − 3.65**	** < 0.001**	** − 6.22**	** − 8.85; − 3.60**	** < 0.001**	** − 3.08**	** − 5.36; − 0.79**	**0.008**			
Neglect	** − 5.16**	** − 7.69, − 2.63**	** < 0.001**	** − 5.11**	** − 7.65; − 2.57**	** < 0.001**	** − 2.67**	** − 4.91; − 0.44**	**0.019**			
FEP patients	*N* = 755			*N* = 755			*N* = 754			*N* = 705		
Overall maltreatment*	** − **2.17	** − **4.78, − 0.45	0.104	** − **2.09	** − **4.71; 0.53	0.118	** − **0.41	** − **2.75; 1.92	0.729	** − **0.07	** − **2.50; 2.35	0.954
Abuse	** − **0.42	** − **2.70; 2.61	0.975	0.01	** − **2.65; 2.66	0.996	1.57	** − **0.78; 3.92	0.191	2.25	** − **0.20; 4.70	0.071
Neglect	** − 2.90**	** − 5.50, − 0.29**	**0.029**	** − 2.80**	** − 5.42; − 0.19**	**0.036**	** − **0.96	** − **3.28; 1.38	0.422	** − **0.63	** − **3.06; 1.80	0.613

Standardized models	Model 1			Model 2			Model 3			Model 4		
	*Beta*	95% CI	*p*	*Beta*^a^	95% CI	*p*	*Beta*^a+b^	95% CI	*p*	*Beta*^a+b+c^	95% CI	*p*
Controls	*N* = 1219			*N* = 1219			*N* = 1216					
Overall maltreatment*	** − 0.30**	** − 0.44; − 0.15**	** < 0.001**	** − 0.30**	** − 0.44; − 0.15**	** < 0.001**	** − 0.13**	** − 0.26; − 0.00**	**0.045**			
Abuse	** − 0.35**	** − 0.50; − 0.21**	** < 0.001**	** − 0.35**	** − 0.50; − 0.20**	** < 0.001**	** − 0.17**	** − 0.30; − 0.04**	**0.008**			
Neglect	** − 0.29**	** − 0.43; − 0.15**	** < 0.001**	** − 0.29**	** − 0.43; − 0.15**	** < 0.001**	** − 0.15**	** − 0.28; − 0.03**	**0.019**			
FEP patients	*N* = 755			*N* = 755			*N* = 754			*N* = 705		
Overall maltreatment*	** − **0.12	** − **0.26; 0.03	0.104	** − **0.12	** − **0.26; 0.03	0.118	** − **0.02	** − **0.15; 0.11	0.729	** − **0.00	** − **0.14; 0.13	0.954
Abuse	** − **0.00	** − **0.15; 0.15	0.975	** − **0.00	** − **0.15; 0.15	0.996	0.09	** − **0.04; 0.22	0.191	0.13	** − **0.01; 0.26	0.071
Neglect	** − 0.16**	** − 0.30; − 0.02**	**0.029**	** − 0.16**	** − 0.30; − 0.01**	**0.036**	** − **0.05	** − **0.18; 08	0.422	** − **0.04	** − **0.17; 0.10	0.613

*CI* confidence intervals, *FEP* first-episode psychosis, *IQ* intelligence quotient

^a^Adjusted for family history of psychosis

^b^Adjusted for sex, age, ethnicity, education, study country, and lifetime cannabis use

^c^Adjusted for antipsychotic treatment

*Exposure to overall childhood maltreatment, and separately childhood abuse, and childhood neglect were defined as mean CTQ > 80th percentile of the control group; significant associations (*p* < 0.05) are shown in bold type

FH was more prevalent among FEP cases than among controls (OR = 3.10 [2.23, 4.31], *p* < 0.001). FH was associated with childhood abuse in the control group (OR = 1.94 [1.09, 3.47], *p* = 0.025) and with childhood neglect in both groups (controls: OR = 1.88 [1.06, 3.32], *p* = 0.030; FEP: OR = 1.54 [1.02, 2.33], *p* = 0.039). FH was not strongly associated with IQ in either group (controls: *B* =  − 1.95 [− 6.52, 2.62], *p* = 0.403; FEP: B =  − 2.10 [− 5.81, 1.62], *p* = 0.269) (see Supplementary Table 3). Multivariable analyses suggested that including FH in the model did not attenuate the association between childhood maltreatment and IQ in cases or in controls (see Table 1).

### Association between childhood maltreatment, SZ-PRS, and IQ

Eight-hundred and fifty community controls and 488 FEP patients (i.e., 66.2% and 58.9% of the original sample, respectively) had complete measures of childhood maltreatment, IQ, and SZ-PRS (see Supplementary Table 4 for sample demographics).

In the control group, a 4–5-point decrease in IQ was associated with childhood maltreatment (*B* =  − 5.11 [− 8.12, − 2.10], *p* < 0.001), abuse (*B* =  − 4.27 [− 7.12, − 1.41], *p* = 0.003), and neglect (*B* =  − 5.18 [− 8.09, − 2.28], *p* < 0.001). In the FEP group, the association between childhood maltreatment (*B* =  − 1.86 [− 5.10, 1.39], *p* = 0.261), abuse (*B* =  − 0.51 [− 3.75, 2.73], *p* = 0.757), neglect (*B* =  − 2.13 [− 5.34; 1.09], *p* = 0.194), and IQ were of a similar size to those detected in the analyses of the full sample, although failed to reach significance.

SZ-PRS were higher in FEP cases than in controls (*B* = 0.54 [0.43, 0.65], *p* < 0.001). In the case group, SZ-PRS was modestly associated with childhood maltreatment (OR = 1.32 [1.03, 1.68], *p* = 0.027) and with childhood neglect (OR = 1.26 [0.99, 1.60], *p* = 0.060). However, in the control group, no associations were found (SZ-PRS and childhood maltreatment OR = 1.00 [0.78, 1.27], *p* = 0.970; abuse OR = 1.00 [0.79, 1.26], *p* = 0.974; neglect OR = 0.98 [0.77, 1.25], *p* = 0.890).

In the control group, higher SZ-PRS was marginally associated with lower IQ (*B* =  − 1.42 [− 2.93, 0.92], *p* = 0.066), as well as in the case group (*p* = 0.180). Multivariable analyses suggest that including SZ-PRS did not reduce the childhood maltreatment-IQ association in either the case or control group (see Supplementary Table 5).

## Discussion

This study found that although FH and SZ-PRS were associated with a greater likelihood of reporting childhood maltreatment (indicating a potential gene-environment correlation), they were not so strongly associated with IQ, and did not attenuate the crude association between childhood maltreatment and IQ in either FEP cases or unaffected controls. These findings are consistent with previous studies suggesting that, although FH is three-to-seven times more common among patients compared to controls [31, 32], its effect on psychosis risk seems relatively independent from the effect of childhood maltreatment, and vice versa [33, 34].

In the subset of European ancestry participants with complete information about childhood maltreatment, IQ, and SZ-PRS, there was evidence of an association between childhood maltreatment and poor cognition, which failed to reach statistical significance among FEP cases. This is different from our previous analysis of this dataset [2] and might be due either to insufficient power, or to the exclusion of non-European ancestry FEP patients (i.e., potentially the relationship between childhood maltreatment and IQ might be more evident among non-European ancestry FEP patients), or to both. Among both cases and controls, SZ-PRS was weakly associated with IQ and did not attenuate the crude association between childhood maltreatment and IQ. This suggests that childhood maltreatment and genetic liability for schizophrenia may be independently associated with poor cognitive functioning.

In conclusion, our findings suggest that two measures of genetic liability for psychosis (FH and SZ-PRS) cannot account for the lower levels of cognition found among adults maltreated in childhood. Future research should use large longitudinal studies to prospectively investigate the impact of genetic liability and childhood maltreatment on different stages of cognitive development. Presently, the reason why the association between childhood adversity and cognition in patients with a psychotic disorder is weaker than in controls remains to be elucidated.

## Supplementary Information

Below is the link to the electronic supplementary material.

Supplementary file1 (DOCX 87 KB)

## References

[1] Vargas T, Lam PH, Azis M (2019). Childhood trauma and neurocognition in adults with psychotic disorders: a systematic review and meta-analysis. Schizophr Bull.

[2] Sideli L, Schimmenti A, La Barbera D (2022). Childhood maltreatment, educational attainment, and IQ: findings from a multicentric case-control study of first-episode psychosis (EU-GEI). Schizophr Bull.

[3] Aas M, Djurovic S, Athanasiu L (2012). Serotonin transporter gene polymorphism, childhood trauma, and cognition in patients with psychotic disorders. Schizophr Bull.

[4] Aas M, Haukvik UK, Djurovic S (2013). BDNF val66met modulates the association between childhood trauma, cognitive and brain abnormalities in psychoses. Prog Neuro-Psychopharmacol Biol Psychiatry.

[5] Green MJ, Chia TY, Cairns MJ (2014). Catechol-O-methyltransferase (COMT) genotype moderates the effects of childhood trauma on cognition and symptoms in schizophrenia. J Psychiatr Res.

[6] Green MJ, Raudino A, Cairns MJ (2015). Do common genotypes of FK506 binding protein 5 (FKBP5) moderate the effects of childhood maltreatment on cognition in schizophrenia and healthy controls?. J Psychiatr Res.

[7] Danese A, Moffitt TE, Arseneault L (2017). The origins of cognitive deficits in victimized children: implications for neuroscientists and clinicians. Am J Psychiatry.

[8] van Os J, Marsman A, van Dam D (2017). Evidence that the impact of childhood trauma on IQ is substantial in controls, moderate in siblings, and absent in patients with psychotic disorder. Schizophr Bull.

[9] van Os J, van Der Steen Y, Islam MA (2017). Evidence that polygenic risk for psychotic disorder is expressed in the domain of neurodevelopment, emotion regulation and attribution of salience. Psychol Med.

[10] Santesteban-Echarri O, Sandel D, Liu L (2022). Family history of psychosis in youth at clinical high risk: a replication study. Psychiatry Res.

[11] Murray GK, Lin T, Austin J (2021). Could polygenic risk scores be useful in psychiatry? A review. JAMA Psychiat.

[12] Wray NR, Lin T, Austin J (2021). From basic science to clinical application of polygenic risk scores: a primer. JAMA Psychiat.

[13] Van Os J, Pries LK, Delespaul P (2020). Replicated evidence that endophenotypic expression of schizophrenia polygenic risk is greater in healthy siblings of patients compared to controls, suggesting gene-environment interaction. The EUGEI study Psychol Med.

[14] Hubbard L, Tansey KE, Rai D (2016). Evidence of common genetic overlap between schizophrenia and cognition. Schizophr Bull.

[15] Hagenaars SP, Harris SE, Davies G (2016). Shared genetic aetiology between cognitive functions and physical and mental health in UK Biobank (*N*=112 151) and 24 GWAS consortia. Mol Psychiatry.

[16] Legge SE, Cardno AG, Allardyce J (2021). Associations between schizophrenia polygenic liability, symptom dimensions, and cognitive ability in schizophrenia. JAMA Psychiat.

[17] Richards AL, Pardiñas AF, Frizzati A (2020). The relationship between polygenic risk scores and cognition in Schizophrenia. Schizophr Bull.

[18] Lencz T, Knowles E, Davies G (2014). Molecular genetic evidence for overlap between general cognitive ability and risk for schizophrenia: a report from the Cognitive Genomics consorTium (COGENT). Mol Psychiatry.

[19] Woolway GE, Smart SE, Lynham AJ (2022). Schizophrenia polygenic risk and experiences of childhood adversity: a systematic review and meta-analysis. Schizophr Bull.

[20] Van Os J, Rutten BPBP, Myin-Germeys I (2014). Identifying gene-environment interactions in schizophrenia: contemporary challenges for integrated, large-scale investigations. Schizophr Bull.

[21] Gayer-Anderson C, Jongsma HE, Di Forti M (2020). The EUropean Network of National Schizophrenia Networks Studying Gene-Environment Interactions (EU-GEI): incidence and First-Episode Case-Control Programme. Soc Psychiatry Psychiatr Epidemiol.

[22] Jongsma HE, Gayer-Anderson C, Lasalvia A (2018). Treated incidence of psychotic disorders in the multinational EU-GEI study. JAMA Psychiat.

[23] Blyler CR, Gold JM, Iannone VN, Buchanan RW (2000). Short form of the WAIS-III for use with patients with schizophrenia. Schizophr Res.

[24] Velthorst E, Levine SZ, Henquet C (2013). To cut a short test even shorter: reliability and validity of a brief assessment of intellectual ability in Schizophrenia—a control-case family study. Cogn Neuropsychiatry.

[25] Velthorst E, Mollon J, Murray RM (2021). Cognitive functioning throughout adulthood and illness stages in individuals with psychotic disorders and their unaffected siblings. Mol Psychiatry.

[26] NIMH Genetics Initiative (1992) Family interview for genetic studies (FIGS). National Institute of Mental Health, Rockville

[27] Quattrone D, Reininghaus U, Richards AL (2021). The continuity of effect of schizophrenia polygenic risk score and patterns of cannabis use on transdiagnostic symptom dimensions at first-episode psychosis: findings from the EU-GEI study. Transl Psychiatry.

[28] Ferraro L, Quattrone D, La Barbera D (2022). First-episode psychosis patients who deteriorated in the premorbid period do not have higher polygenic risk scores than others: a cluster analysis of EU-GEI data. Schizophr Bull.

[29] Tripoli G, Quattrone D, Ferraro L (2022). Facial emotion recognition in psychosis and associations with polygenic risk for schizophrenia: findings from the multi-center EU-GEI case-control study. Schizophr Bull.

[30] Bernstein DP, Ahluvalia T, Pogge D, Handelsman L (1997). Validity of the childhood trauma questionnaire in an adolescent psychiatric population. J Am Acad Child Adolesc Psychiatry.

[31] Trotta A, Di FM, Iyegbe C (2015). Familial risk and childhood adversity interplay in the onset of psychosis. BJPsych Open.

[32] Fisher HL, McGuffin P, Boydell J (2014). Interplay between childhood physical abuse and familial risk in the onset of psychotic disorders. Schizophr Bull.

[33] van Winkel R, van Nierop M, Myin-Germeys I, van Os J (2013). Childhood trauma as a cause of psychosis: linking genes, psychology, and biology. Can J Psychiatry.

[34] Sideli L, Murray RM, Schimmenti A (2020). Childhood adversity and psychosis: a systematic review of bio-psycho-social mediators and moderators. Psychol Med.

