# Supplementary material for: The relationship between genetic liability, childhood maltreatment, and IQ: findings from the EU-GEI multicentric case–control study
Source: Soc Psychiatry Psychiatr Epidemiol. 2023 Jun 19;58(10):1573–80. doi: 10.1007/s00127-023-02513-0 (PMC10460355; doi:10.1007/s00127-023-02513-0)
Supplement: Supplementary file 1 — Supplementary file1 (DOCX 87 KB) [file 127_2023_2513_MOESM1_ESM.docx]

**The relationship between genetic liability, childhood maltreatment, and IQ: findings from the multicentric EU-GEI case-control study**

**Suppl. Information 1: Participants and procedure**

The EU-GEI study was a multicentric case-control incident study, carried out between May 2010 and April 2015 across France, Holland, Italy, Spain, UK, and Brazil. The study was approved by the Internal Review Boards of the study centres and written informed consent were provided by study participants [1, 2].

People with first-episode psychosis (FEP) were considered eligible if they were incident cases of psychosis, aged 18-64 and resident in the study catchment areas. People with FEP were included if they have approached mental health services for the first time during the study period for a diagnosis of psychotic disorder (ICD-10 diagnoses: F20–F33), neither secondary to acute intoxication (ICD-10: F1X.5) nor to medical condition (ICD-10: F09), and not previously treated with antipsychotics. Diagnoses of FEP were established according to ICD-10 criteria [3], by trained researchers using the Operational Criteria Checklist algorithm, OPCRIT [4] [5]. Clinical diagnoses were used in the few cases in which OPCRIT was not available (12.1%).

Community controls were considered eligible if they were aged 18-64 and resident in the same catchment areas as people with FEP. Community controls were included if they have never received diagnosis or treatment for psychotic disorders. To ensure that controls were representative of the same population as the cases in terms of sex, age, and ethnicity, the study used a combination of random and quota sampling strategies [5, 6].

**Suppl. Information 2: Measures of childhood maltreatment and cognition**

Childhood maltreatment was assessed using the Childhood Trauma Questionnaire (CTQ) [7] a widely used, psychometrically robust, self-report instrument assessing the frequency of physical, sexual, and emotional abuse, and physical and emotional neglect prior to age 18. An overall “childhood maltreatment” score, and separate “childhood abuse” and “childhood neglect” scores were calculated on the basis of the mean score of the respective items, ranging from 1 [never] to 5 [very often]. Consistently with previous studies [8, 9], three dichotomous variables for overall childhood maltreatment, abuse, and neglect were calculated using the 80th percentile of the control group as a cut-off value. These cut-off were identical to those used in the original study (overall childhood maltreatment = 1.60, childhood abuse =1.40, childhood neglect = 1.90) [9]. The average exposure to childhood maltreatment in the patients (mean =1.66, SD=0.55) and the control group (mean =1.37, SD=0.43) were similar to those observed in the original study (patients: mean =1.67, SD=0.57; controls: mean =1.37, SD=0.43) and in the GROUP study (patients: mean =1.61, SD=0.50; controls: mean =1.34, SD=0.36) [8].

Cognitive functioning was estimated from overall Intelligence Quotient (IQ) assessed using an abbreviated version of the Wechsler Adult Intelligence Scale (WAIS-III) [10], including selected items of the following subtests: digit symbol coding (a measure of processing speed), arithmetic (working memory), block design (visuospatial processing), and information (verbal knowledge). The administration and scoring procedure of the abbreviated version have been previously described and psychometrically validated [11, 12]. The mean IQ in the patients (mean = 85.82, SD= 18.08) and the control group (mean = 103.24, SD= 17.72) were similar to those observed in the original study (patients: mean = 85.04, SD= 18.18; controls: mean = 102.97, SD= 17.63).

Information about socio-demographic characteristic (i.e., sex, age, country, and ethnicity, defined as white vs. non-white) were collected using the MRC sociodemographic questionnaire [13]. Lifetime cannabis use was defined as having ever used cannabis lifetime and assessed using the Cannabis Experience Questionnaire (CEQmv) [14]. Information about current use of antipsychotics at the time of the interview (defined as none vs. one vs. more than one antipsychotic medications) was collected from clinical records.

**Suppl. Information 3: Measures of genetic liability**

Family history of psychosis was assessed by trained researchers using the Family Interview for Genetic Studies [15]. The FIGS is a semi-structured interview which assesses history of mental disorders amongst first-degree relatives, namely participants’ biological parents, siblings, and children. First, the family pedigree diagram was drafted. Then, history of mental disorders among first-degree relatives was explored using screening questions. In case of positive response to the screening questions, supplementary modules were administered to ascertain any current or past specific disorder (e.g., psychosis, depression, or mania). For this study, only a dichotomous variable of family history of psychosis was used.

Study participants were genotyped at the MRC Centre for Neuropsychiatric Genetics and Genomics in Cardiff (UK) using a custom Illumina HumanCoreExome-24 BeadChip genotyping array, which covers 570,038 genetic variants. Genetic variants with call rate <98% or with Hardy-Weinberg Equilibrium p-value < 1e-6 and samples with call rate <98% were excluded from the dataset. To reduce the variability due to genetic ancestry, analyses were restricted to participants of European ancestry, determined by: merging the EU-GEI sample with the 1000 Genome project sample phase 3, building ancestry Principal Components (PCs) of the overlapping SNPs, and applying k-mean clustering [16]. To account for population stratification within the European EU-GEI sample, a Principal Component Analysis was carried out on pruned linkage disequilibrium variants, and the first 10 ancestry principal components (PCs) were subsequently included as covariates in the genetic analyses. SZ-PRS was calculated using a shrinkage method in PRS-CS [17], based on the summary statistics from the latest SZ-GWAS from where the current EU-GEI sample was excluded [18].

**References**

1. Van Os J, Rutten BPBP, Myin-Germeys I, et al (2014) Identifying gene-environment interactions in schizophrenia: Contemporary challenges for integrated, large-scale investigations. Schizophr Bull 40:729–736. https://doi.org/10.1093/schbul/sbu069

2. Gayer-Anderson C, Jongsma HE, Di Forti M, et al (2020) The EUropean Network of National Schizophrenia Networks Studying Gene–Environment Interactions (EU-GEI): Incidence and First-Episode Case–Control Programme. Soc Psychiatry Psychiatr Epidemiol 55:. https://doi.org/10.1007/s00127-020-01831-x

3. World Health Organization (1992) The ICD-10 classification of mental and behavioural disorders: clinical descriptions and diagnostic guidelines. World Health Organization, Geneva

4. McGuffin P, Farmer A, Harvey I (1991) A polydiagnostic application of operational criteria in studies of psychotic illness: Development and reliability of the OPCRIT system. Arch Gen Psychiatry 48:764–770. https://doi.org/10.1001/archpsyc.1991.01810320088015

5. Jongsma HE, Gayer-Anderson C, Lasalvia A, et al (2018) Treated incidence of psychotic disorders in the multinational EU-GEI study. JAMA Psychiatry 75:. https://doi.org/10.1001/jamapsychiatry.2017.3554

6. Gayer-Anderson C, Jongsma HE, Di Forti M, et al (2020) The EUropean Network of National Schizophrenia Networks Studying Gene–Environment Interactions (EU-GEI): Incidence and First-Episode Case–Control Programme. Soc Psychiatry Psychiatr Epidemiol 55:645–657. https://doi.org/10.1007/s00127-020-01831-x

7. Bernstein DP, Ahluvalia T, Pogge D, Handelsman L (1997) Validity of the childhood trauma questionnaire in an adolescent psychiatric population. J Am Acad Child Adolesc Psychiatry 36:340–348. https://doi.org/10.1097/00004583-199703000-00012

8. van Os J, Marsman A, van Dam D, et al (2017) Evidence that the impact of childhood trauma on IQ is substantial in controls, moderate in siblings, and absent in patients with psychotic disorder. Schizophr Bull 43:316–324. https://doi.org/10.1093/schbul/sbw177

9. Sideli L, Schimmenti A, La Barbera D, et al (2022) Childhood maltreatment, educational attainment, and IQ: Findings from a multicentric case-control study of First-episode Psychosis (EU-GEI). Schizophr Bull 48:575–589. https://doi.org/10.1093/schbul/sbac004

10. Blyler CR, Gold JM, Iannone VN, Buchanan RW (2000) Short form of the WAIS-III for use with patients with schizophrenia. Schizophr Res 46:209–215. https://doi.org/10.1016/S0920-9964(00)00017-7

11. Velthorst E, Levine SZ, Henquet C, et al (2013) To cut a short test even shorter: Reliability and validity of a brief assessment of intellectual ability in Schizophrenia - A control-case family study. Cogn Neuropsychiatry 18:574–593. https://doi.org/10.1080/13546805.2012.731390

12. Velthorst E, Mollon J, Murray RM, et al (2021) Cognitive functioning throughout adulthood and illness stages in individuals with psychotic disorders and their unaffected siblings. Mol Psychiatry. https://doi.org/10.1038/s41380-020-00969-z

13. Mallet R (1997) Sociodemographic schedule. Section of Social Psychiatry, Institute of Psychiatry, London

14. Di Forti M, Sallis H, Allegri F, et al (2014) Daily use, especially of high-potency cannabis, drives the earlier onset of psychosis in cannabis users. Schizophr Bull 40:1509–1517. https://doi.org/10.1093/schbul/sbt181

15. NIMH Genetics Initiative (1992) Family Interview for Genetic Studies (FIGS). National Institute of Mental Health, Rockville, MD

16. Quattrone D, Reininghaus U, Richards AL, et al (2021) The continuity of effect of schizophrenia polygenic risk score and patterns of cannabis use on transdiagnostic symptom dimensions at first-episode psychosis: findings from the EU-GEI study. Transl Psychiatry 11:1–10. https://doi.org/10.1038/s41398-021-01526-0

17. Ge T, Chen CY, Ni Y, et al (2019) Polygenic prediction via Bayesian regression and continuous shrinkage priors. Nat Commun 10:1–10. https://doi.org/10.1038/s41467-019-09718-5

18. Trubetskoy V, Pardiñas AF, Qi T, et al (2022) Mapping genomic loci implicates genes and synaptic biology in schizophrenia. Nature 604:502–508. https://doi.org/10.1038/s41586-022-04434-5

**Supplementary Table 1: Demographic characteristics of FEP patients and controls with information about family history of psychosis**

| **Variable** | **FEP (n=** **755)**  ***n* (%)** | **Controls (n=** **1219)**  ***n* (%)** | ***χ^2^*/ *t* (*df*)** | ***p*** |
| --- | --- | --- | --- | --- |
| **Sex, Male** | 465 (61.6) | 580 (47.6) | 36.73 (1) | <.001 |
| **Age, *M* (*SD*)** | 30.59 (10.40) | 36.06 (13.08) | 9.74 (1970) | <.001 |
| **Ethnicity, Non-white** | 250 (33.1) | 244 (20.0) | 42.62 (1) | <.001 |
| **Education**   - **No qualification** - **Compulsory** - **Tertiary** - **Job related** - **University** - **Post-degree** | 109 (14.4)  201 (26.6)  186 (24.6)  133 (17.6)  91 (12.1)  35 (4.6) | 53 (4.3)  160 (13.1)  334 (27.4)  199 (16.3)  291 (23.9)  182 (14.9) | 184.69 (5) | <.001 |
| **Country**   - **UK** - **Holland** - **Spain** - **France** - **Italy** - **Brazil** | 137 (18.1)  151 (20.0)  161 (21.3)  59 (7.8)  95 (12.6)  152 (20.1) | 276 (22.6)  190 (15.6)  192 (15.8)  136 (11.2)  154 (12.6)  271 (22.2) | 24.09 (5) | <.001 |
| **Lifetime cannabis use** | 749  494 (66.0) | 1213  586 (48.3) | 58.26 (1) | <.001 |
| **Antipsychotic treatment**   - **More than one** - **One** - **None** | 755  105 (13.9)  163 (21.6)  438 (58.0) |  |  |  |

FEP: first-episode psychosis; df: degrees of freedom; M: mean; SD: standard deviation.

**Supplementary Table 2: Childhood maltreatment, IQ, and family history of psychosis of included FEP patients and controls**

| **Variable** | **FEP (n=** **755)**  ***n* (%)** | **Controls (n=** **1219)**  ***n* (%)** | ***t / χ^2^*(df)** | ***P*** | **OR/ b [95% CI]** | ***p*** |
| --- | --- | --- | --- | --- | --- | --- |
| **Maltreatment exposure ***   - **Overall Childhood Maltreatment, n (%)** - **abuse, n (%)** - **neglect, n (%)** | 318 (42.1)  291 (38.5)  322 (42.6) | 221 (18.1)  209 (17.1)  229 (18.8) | 135.17 (1)  112.87 (1)  131.95 (1) | <.001  <.001  <.001 | 3.29 [2.68, 4.03]  3.03 [2.45, 3.74]  3.22 [2.62, 3.94] | <.001  <.001  <.001 |
| **IQ, *M* (*SD*)** | 85.82 (18.08) | 103.24 (17.72) | 21.06 (1970) | <.001 | 17.94 [16.38, 19.50] | <.001 |
| **Family history of psychosis** | 106 (14.0) | 61 (5.0) | 49.15 (1) | <.001 | 3.10 [2.23, 4.31] | <.001 |

CI: confidence intervals; CTQ: Childhood Trauma Questionnaire; df: degrees of freedom; FEP: first-episode psychosis; IQ: intelligence quotient; M: Mean; OR: odds ratio; SD: standard deviation. *Exposure to overall childhood maltreatment, and separately childhood abuse, and childhood neglect were defined as mean CTQ >80^th^ percentile of the control group.

**Supplementary Table 3: Association between childhood maltreatment, family history of psychosis, and IQ among cases and controls**

| **Maltreatment exposure*** | **Unexposed**  ***mean IQ (SD)*** | **Exposed**  ***mean iq (SD)*** | ***t* (*df*)** | ***p*** |
| --- | --- | --- | --- | --- |
| **Controls (*N*=1219)** |  |  |  |  |
| **Childhood maltreatment (998 vs. 221)**  **Abuse (1010 vs. 209)**  **Neglect (990 vs. 229)** | 104.20 (17.54)  104.32 (17.74)  104.21 (17.39) | 98.91 (17.94)  98.05 (16.71)  99.05 (18.56) | 4.04 (1217)  4.70 (1217)  4.00 (1217) | <.001  <.001  <.001 |
| **Family history for psychosis (1158 vs. 61)** | 103.34 (17.7) | 101.39 (18.09) | 0.84 (1217) | .403 |
| **FEP Patients (*N*=755)** |  |  |  |  |
| **Childhood maltreatment (437 vs. 318)**  **Abuse (464 vs. 291)**  **Neglect (433 vs. 322)** | 86.73 (18.35)  85.84 (18.18)  87.06 (18.37) | 84.57 (17.65)  85.79 (17.95)  84.16 (17.58) | 1.63 (753)  0.03 (753)  2.18 (753) | .104  .975  .029 |
| **Family history for psychosis (649 vs. 106)** | 86.11 (18.5) | 84.02 (15.36) | 1.10 (753) | .269 |

CI: confidence intervals; CTQ: Childhood Trauma Questionnaire; df: degrees of freedom; FEP: first-episode psychosis; IQ: intelligence quotient; M: Mean; OR: odds ratio; SD: standard deviation. *Exposure to overall childhood maltreatment, and separately childhood abuse, and childhood neglect were defined as mean CTQ >80^th^ percentile of the control group.

**Supplementary Table 4: Demographic characteristics of included FEP patients and controls with Polygenic Risk Score for schizophrenia**

| **Variable** | **FEP (n=** **488)**  ***n* (%)** | **Controls (n=** **850)**  ***n* (%)** | ***χ^2^*/ *t* (*df*)** | ***p*** |
| --- | --- | --- | --- | --- |
| **Sex, Male** | 310 (63.5) | 402 (47.3) | 32.80 (1) | <.001 |
| **Age, *M* (*SD*)** | 31.43 (10.85) | 37.35 (13.27) | 8.38 (1334) | <.001 |
| **Ethnicity, Non-white** | 80 (16.4) | 78 (9.2) | 15.44 (1) | <.001 |
| **Education**   - **No qualification** - **Compulsory** - **Tertiary** - **Job related** - **University** - **Post-degree** | 68 (13.9)  124 (25.4)  122 (25.0)  90 (18.4)  58 (11.9)  26 (5.3) | 32 (3.8)  111 (13.1)  204 (24.0)  149 (17.5)  220 (25.9)  134 (15.8) | 127.57 (5) | <.001 |
| **Country**   - **UK** - **Holland** - **Spain** - **France** - **Italy** - **Brazil** | 70 (14.3)  114 (23.4)  142 (29.1)  36 (7.4)  48 (9.8)  78 (16.0) | 182 (21.4)  146 (17.2)  159 (18.7)  93 (10.9)  78 (9.2)  192 (22.6) | 40.14 (5) | <.001 |
| **Lifetime cannabis use** | 482  331 (68.7) | 846  413 (48.8) | 49.13 (1) | <.001 |
| **Antipsychotic treatment**   - **More than one** - **One** - **None** | 450  69 (15.3)  95 (21.1)  286 (63.6) |  |  |  |

df: degrees of freedom; FEP: first-episode psychosis; M: Mean; SD: standard deviation.

**Supplementary Table 5: Association between childhood maltreatment and IQ controlled for Polygenic Risk Score for schizophrenia and social and clinical confounders**

| **Childhood maltreatment exposure*** | **Model 1** | | | **Model 2** | | | **Model 3** | | | **Model 4** | | |
| --- | --- | --- | --- | --- | --- | --- | --- | --- | --- | --- | --- | --- |
|  | *B* | 95% CI | *p* | *B* ^a^ | 95% CI | *p* | *B*  ^a+b^ | 95% CI | *p* | *B*  ^a+b+c^ | 95% CI | *p* |
| **Controls** | *N*= 573 | | | *N*= 573 | | | *N*= 571 | | |  | | |
| **Overall Maltreatment**  **Abuse**  **neglect** | **-5.11**  **-4.27**  **-5.18** | **-8.12, -2.10**  **-7.12, -1.41**  **-8.09, -2.28** | **<.001**  **.003**  **<.001** | **-4.60**  **-3.42**  **-5.10** | **-7.53, -1.67**  **-6.21, -0.63**  **-7.95, -2.24** | **.002**  **.016**  **<.001** | -1.94  **-**1.32  -2.59 | -4.64, 0.76  -3.86, 1.22  -5.21, -0.03 | .159  .309  .053 |  |  |  |
| **FEP Patients** | *N*= 343 | | | *N*= 343 | | | *N*= 337 | | | *N*=314 | | |
| **Overall Maltreatment**  **Abuse**  **neglect** | -1.86  -0.51  -2.13 | -5.10, 1.39  -3.75, 2.73  -5.34, 1.09 | .261  .757  .194 | -2.18  -0.19  -2.59 | -5.33, .963  -3.33, 2.96  -5.69, 0.51 | **.**173  **.**907  **.**102 | -1.15  -0.42  -1.05 | -4.04, 1.75  -3.26, 2.42  -3.88, 1.78 | .436  .771  .467 | -1.30  -0.53  -1.31 | -4.33, 1.73  -3.52, 2.46  -4.28, 1.67 | .399  .727  .388 |
| **Standardized models** | **Model 1** | | | **Model 2** | | | **Model 3** | | | **Model 4** | | |
| **Controls** | *Beta* | 95% CI | *p* | *Beta* ^a^ | 95% CI | *p* | *Beta*  ^a+b^ | 95% CI | *p* | *Beta*  ^a+b+c^ | 95% CI | *p* |
| **Overall Maltreatment**  **Abuse**  **neglect** | **-.30**  **-.25**  **-.31** | **-.48, -.12**  **-.42, -.08**  **-.48, -.13** | **<.001**  **.003**  **<.001** | **-.27**  **-.20**  **-.30** | **-.44, -.10**  **-.37, -.03**  **-.47, -.13** | **.002**  **.016**  **<.001** | -.11  -.08  -.15 | -.27, .05  -.23, .07  -.31, .00 | .159  .309  .053 |  |  |  |
| **FEP Patients** |  |  |  |  |  |  |  |  |  |  |  |  |
| **Overall Maltreatment**  **Abuse**  **neglect** | -.10  -.03  -.12 | -.28, .08  -.21, .15  -.30, .06 | .261  .757  .194 | -.12  -.01  -.14 | -.30, .05  -.19, .17  -.32, .03 | .173  .907  .102 | -.06  -.02  -.06 | -.23, .10  -.18, .14  -.22, .10 | .436  .771  .467 | -.07  -.03  -.07 | -.24, .10  -.20, .14  -.24, .09 | .399  .727  .388 |

CI: confidence intervals; FEP: first-episode psychosis. ^a^ adjusted for PRS and ancestry principal components (PCA); ^b^ adjusted for sex, age, education, study country, and lifetime cannabis use; ^c^ adjusted for antipsychotic treatment; *exposure to overall childhood maltreatment, and separately childhood abuse, and childhood neglect were defined as mean CTQ >80th percentile of the control group; associations (*p* <.05) are shown in bold type.
